# A Major Facilitator Superfamily Transporter-Mediated Resistance to Oxidative Stress and Fungicides Requires Yap1, Skn7, and MAP Kinases in the Citrus Fungal Pathogen *Alternaria alternata*

**DOI:** 10.1371/journal.pone.0169103

**Published:** 2017-01-06

**Authors:** Li-Hung Chen, Hsieh-Chin Tsai, Pei-Ling Yu, Kuang-Ren Chung

**Affiliations:** 1 Department of Plant Pathology, College of Agriculture and Natural Resources, National Chung-Hsing University, Taichung, Taiwan; 2 Biotechnology Center, NCHU, Taichung, Taiwan; 3 NCHU-UCD Plant and Food Biotechnology Center, NCHU, Taichung, Taiwan; Yonsei University, REPUBLIC OF KOREA

## Abstract

Major Facilitator Superfamily (MFS) transporters play an important role in multidrug resistance in fungi. We report an *AaMFS19* gene encoding a MFS transporter required for cellular resistance to oxidative stress and fungicides in the phytopathogenic fungus *Alternaria alternata*. AaMFS19, containing 12 transmembrane domains, displays activity toward a broad range of substrates. Fungal mutants lacking *AaMFS19* display profound hypersensitivities to cumyl hydroperoxide, potassium superoxide, many singlet oxygen-generating compounds (eosin Y, rose Bengal, hematoporphyrin, methylene blue, and cercosporin), and the cell wall biosynthesis inhibitor, Congo red. *AaMFS19* mutants also increase sensitivity to copper ions, clotrimazole, fludioxonil, and kocide fungicides, 2-chloro-5-hydroxypyridine (CHP), and 2,3,5-triiodobenzoic acid (TIBA). *AaMFS19* mutants induce smaller necrotic lesions on leaves of a susceptible citrus cultivar. All observed phenotypes in the mutant are restored by introducing and expressing a wild-type copy of *AaMFS19*. The wild-type strain of *A*. *alternata* treated with either CHP or TIBA reduces radial growth and formation and germination of conidia, increases hyphal branching, and results in decreased expression of the *AaMFS19* gene. The expression of *AaMFS19* is regulated by the Yap1 transcription activator, the Hog1 and Fus3 mitogen-activated protein (MAP) kinases, the ‘two component’ histidine kinase, and the Skn7 response regulator. Our results demonstrate that *A*. *alternata* confers resistance to different chemicals via a membrane-bound MFS transporter.

## Introduction

Major Facilitator Superfamily (MFS) transporters have been demonstrated to be involved in multidrug resistance in fungi [1,2]. MFS transporters are capable of transporting small molecules in response to ion gradients or function as drug:H^+^ antiporter in microorganisms. Mounting evidence indicates that MFS transporter may also indirectly control membrane potential by changing membrane lipid homeostasis, and regulate internal pH and the stress response machinery in fungi [2]. Many MFS transporters are required for microorganisms to grow under stress conditions. In the budding yeast, MFS transporters containing either a 12- or 14-transmembrane domain have been demonstrated to confer resistance to a wide array of chemicals and drugs [3] and their regulation has been found to be controlled by several stress related transcription factors including Yap1, Msn2, Msn4, and Sfp1 [2]. In phytopathogenic fungi, MFS transporters have been shown to be involved in resistance to toxins and fungicides [4–8].

The tangerine pathotype of *Alternaria alternata* produces a host selective (HS) toxin that kills host cells prior to colonization. The ability to produce the HS toxin is required for *A*. *alternata* pathogenesis [9]. *Alternaria alternata* infection in citrus leaves triggers rapid lipid peroxidation and accumulation of hydrogen peroxide (H_2_O_2_), which eventually leads to cell death [10]. Experiments have demonstrated that the ability to detoxify toxic reactive oxygen species (ROS) is too required for *A*. *alternata* pathogenesis [11–17].

*Alternaria alternata* is capable of detoxifying toxic ROS via multiple regulatory pathways. *A*. *alternata* mutant strains lacking the Yap1 transcription activator, the Hog1 mitogen-activated protein (MAP) kinase, the Ssk1 regulator, the Skn7 response regulator, the NADPH oxidase (Nox), or the Gpx3 glutathione peroxidase all displayed hypersensitivity to oxidants and reduced lesion formation on citrus [11,12,14–18]. Exogenous addition of iron partially rescued H_2_O_2_ sensitivity seen for *Yap1*, *Hog1*, *Skn7*, and *Nox* mutants [19], indicating the important role of iron uptake in ROS resistance. This could be attributable to the fact that iron is an important cofactor for antioxidant activities and that iron itself could promote ROS detoxification through a non-enzymatic mechanism. Further studies revealed that the expression of the non-ribosomal peptide synthetase coding gene (*Nps6*) and the production of siderophore were regulated by Yap1, Hog1, and Nox, but not by Skn7 [15]. The ability to synthesize siderophore via the Nps6 and to chelate iron has also been demonstrated to be required for ROS detoxification and fungal pathogenesis. Although H_2_O_2_ sensitivity seen in the *Skn7* mutant could be resorted by addition of iron, Skn7 apparently could regulate non-siderophore iron acquisition. Experiments have also demonstrated that mutational inactivation of *Yap1* or *Skn7* resulted in fungal strains that had lower glutathione reductase, catalase, glutathione peroxidase, superoxide dismutase (SOD), glutathione-S-transferase, and ligninolytic peroxidase activities. In addition, the glutathione system played a vital role in ROS detoxification. The expression of the Gpx3 glutathione peroxidase gene has been shown to be coordinately regulated by Yap1, Hog1, and Nox and inactivation of *Gpx3* results in decreased sensitivity to oxidants. Taken collectively, it is tempting to speculate that low-level H_2_O_2_ generated by Nox may likely act as a signaling molecule to activate transcriptional expression and/or nuclear localization of Yap1, Hog1, Skn7, and perhaps many other regulators, which leads to the further activation of downstream genes under oxidative stress conditions in *A*. *alternata*.

Yap1 is a leucine zipper-containing transcriptional activator, which has been demonstrated to be responsible for transcriptional activation of genes involved in ROS detoxification as well as drug and heavy metal resistance in fungi [20]. Yap1 has also been known to act as an important regulator of major facilitator superfamily (MFS) gene expression in yeasts [21–24]. In addition to ROS sensitivity, *A*. *alternata* strains lacking *Yap1* or *Hog1* were hypersensitive to 2-chloro-5-hydroxypyridine (CHP) and 2,3,5-triiodobenzoic acid (TIBA). The toxicity of CHP or TIBA to *A*. *alternata* remains to be determined.

Suppressive subtractive hybridization had identified two genes encoding putative major facilitator superfamily (MFS) transporters that were co-ordinately regulated by the Yap1 transcription regulator in *A*. *alternata* [10]. In the present study we report a functional characterization of a 12-spanner MFS transporter to explore its roles in resistance to oxidants and fungicides, and in virulence. We also determine the toxic effects of CHP or TIBA to *A*. *alternata*.

## Results

### Characterization of an *AaMFS19* gene encoding a major facilitator superfamily transporter (MFS)

The *A*. *alternata MFS* gene (*AaMFS19*, accession # GS597470) encoding a major facilitator superfamily transporter was originally identified from the wild-type cDNA library after subtraction with that of a *Yap1* mutant. Alignment of *AaMFS19* sequence with other fungal MFS transporter sequences in databases available at the National Center for Biotechnology Information (NCBI) revealed that *AaMFS19* was predicted to contain a 1558-bp open reading frame separated by three small introns (61, 55, and 56 bp) that encodes a polypeptide of 461 amino acids. Hydropathy analysis revealed that AaMFS19 contains 12 putative transmembrane domains. The AaMFS19 protein has a predicted molecular mass of 50.2 kDa and a predicted pI of 9.26.

### *AaMFS19* is required for tolerance to oxidants, fungicides and xenobiotics

**Fig 1:**
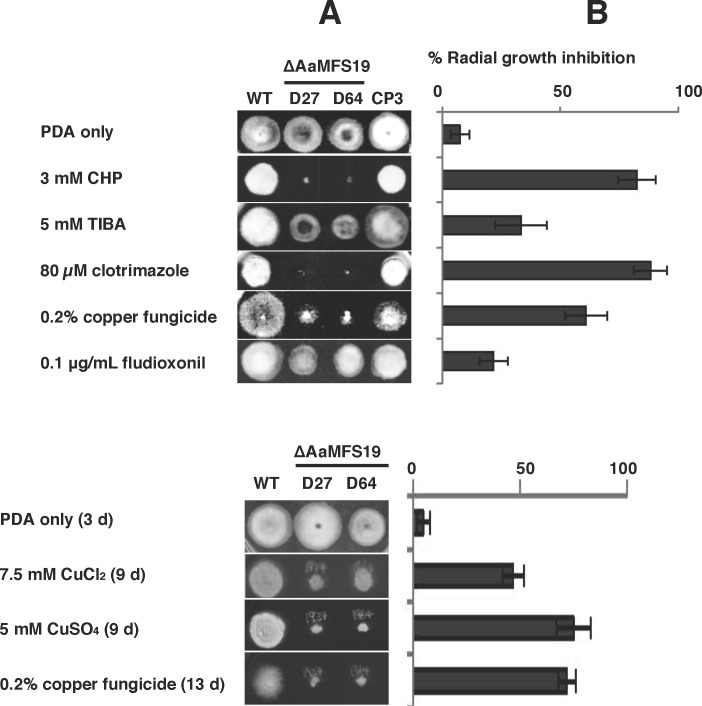
The *Alternaria alternata* major facilitator superfamily transporter (AaMFS19) confers resistance to xenobiotics. **(A)** Images of the wild-type (WT), two ΔAaMFS19 deletion mutants (D27 and D63), and the CP3 complementation strain grown on potato dextrose agar (PDA) amended with 2-chloro-5-hydroxypyridine (CHP), 2,3,5-triiodobenzoic acid (TIBA), fungicides and other chemicals as indicated. Only representative replicates are shown. **(B)** Quantitative analysis of chemical sensitivity. Sensitivity indicated by percentage growth reduction was calculated as a cumulative percentage of growth of wild-type and AaMFS19 mutant strains grown on the same plate. The data presented are the mean and standard deviation of two independent experiments with four biological replicates.

**Fig 2:**
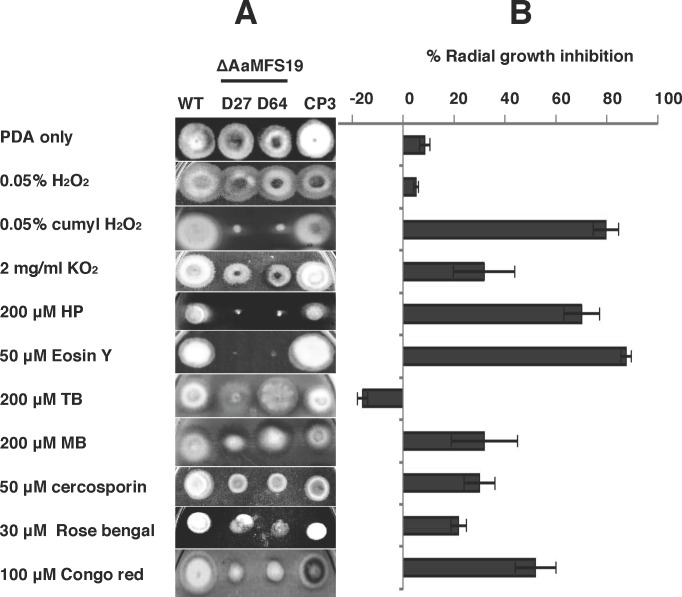
The *Alternaria alternata* major facilitator superfamily transporter (AaMFS19) confers resistance to oxidants and cell wall destructing agents. **(A)** Images of the wild-type (WT), two ΔAaMFS19 deletion mutants (D27 and D63), and the CP3 complementation strain grown on potato dextrose agar (PDA) amended with hydrogen peroxides (H_2_O_2_ and cumyl H_2_O_2_), potassium superoxide (KO_2_), single oxygen-generating compounds, eosin Y, rose Bengal, hematoporphyrin (HP), toluidine blue (TB), methylene blue (MB), and cercosporin, and the cell wall-destructing agent, Congo red. Only representative replicates are shown. **(B)** Quantitative analysis of chemical sensitivity. Sensitivity indicated by percentage growth reduction was calculated as a cumulative percentage of growth of wild-type and AaMFS19 mutant strains grown on the same plate. The data presented are the mean and standard deviation of two independent experiments with four biological replicates.

Previous studies have demonstrated that *A*. *alternata* mutant impaired for the stress-responsive transcription regulator Yap1 increased hypersensitivity to a wide spectrum of oxidants as well as to both 2,3,5-triiodobenzoic acid (TIBA) and 2-chloro-5-hydroxypyridine (CHP) compared to its progenitor strain [10]. To test whether or not *AaMFS19* plays a role in resistance to oxidants, CHP, and TIBA, *A*. *alternata* mutant defective for *AaMFS19* was created by targeted gene disruption. Using a split marker gene deletion approach, two *A*. *alternata* mutants (D27 and D64) defective at *AaMFS19* locus were identified after screening 5 independently selected transformants by PCR (S1 Fig). The *AaMFS19* impaired mutants reduced radial growth by 5 to 8% compared to wild-type cultured on PDA for 3 days. When culturing on PDA in light, both D27 and D64 strains produced ovoid, dark-pigmented conidia with both cross and longitudinal septae similar to those produced by wild-type. Chemical sensitivity assayed on PDA revealed that both the *AaMFS19* deficient mutants (D27 and D64) were more sensitive to CHP, TIBA, clotrimazole, fludioxonil (a phenylpyrrole fungicide), and copper fungicides, CuCl_2_ (7.5 mM), and CuSO_4_ (5 mM) than wild-type (Fig 1). Both D27 and D64 also increased sensitivity to cumyl H_2_O_2_ and the superoxide-generating compound, potassium superoxide (KO_2_) (Fig 2). Both mutants also increased sensitivity to various singlet oxygen-generating chemicals, including eosin Y, rose Bengal (RB), hematoporphyrin (HP), methylene blue (MB), and cercosporin (Fig 2). Compared with wild-type, both D27 and D64 mutants were more sensitive to the cell wall perturbing agent, Congo red. In contrast, toluidine blue (TB) promoted radial growth of both D27 and D64 strains. All observed phenotypes were restored in the CP3 strain by transforming the D27 mutant protoplasts with a wild-type copy of *AaMFS19*. Both D27 and D64 displayed wild-type sensitivity to H_2_O_2_ (0.05%), diamide (a thiol-oxidizing agent; 2.5 mM), menadione (a superoxide-generating compound; 2 mM), and *tert*-butyl hydroperoxide (0.05%) (S2 Fig).

### Expression of *AaMFS19* is suppressed by xenobiotics

**Fig 3:**
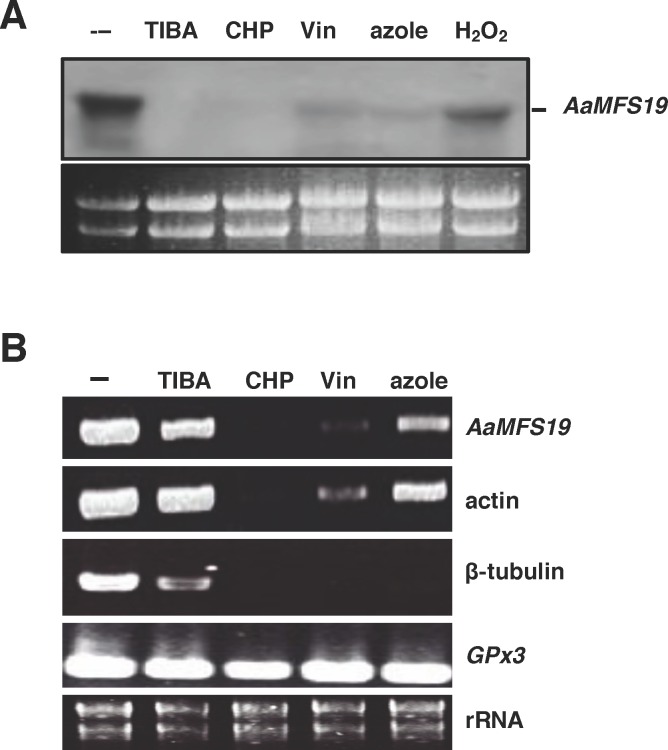
Expression of the *AaMFS19* gene encoding a major facilitator superfamily transporter in *Alternaria alternata*. **(A)** Northern blot analysis of *AaMFS19* in response to 2-chloro-5-hydroxypyridine (CHP), 2,3,5-triiodobenzoic acid (TIBA), vinclozolin (Vin), clotrimazole, and H_2_O_2_. The wild-type strain was grown on a layer of cellophane overlaid on PDA for 3 days and shifted to PDA amended with chemicals as indicated for an additional 24 h. Fungal RNA was purified, electrophoresed on a formaldehyde-containing gel, blotted to membrane, and hybridized with an *AaMFS19*-specific probe. Ethidium bromide (EtBr)-stained rRNA is shown to ensure equal loading of the samples. **(B)** Semi-quantitative reverse transcription PCR analysis of *AaMFS19*, the actin and β-tublin-coding genes, and the *GPx3* gene encoding a glutathione peroxidase. PCR products were visualized on 1.2% agarose gel staining with EtBr.

Northern blot hybridization revealed that expression of *AaMFS19* was down-regulated in the wild-type strain grown on PDA amended with TIBA, CHP, vinclozolin (a dicarboximide fungicide), clotrimazole, or H_2_O_2_ for 3 days (Fig 3A). When wild-type was cultured on PDA for 2 days, shifted to medium amended with TIBA, CHP, vinclozolin, or clotrimazole, and incubated for an additional 24 h, expression of *AaMFS19* was down-regulated as examined by semi-quantitative RT-PCR (Fig 3B). Expression of both actin- and β-tubulin-coding genes was also decreased in the wild-type shifted to medium containing CHP, vinclozolin, or clotrimazole. Shifting to a TIBA-containing medium reduced expression of the β-tubulin-coding gene but did not significantly change the transcript levels of the actin-coding gene in the wild-type. However, the transcript levels of the *GPx3* gene encoding a glutathione peroxidase in the wild-type were not affected by test chemicals.

### Expression of *AaMFS19* is regulated by stress-responsive regulators, MAP kinases and histidine kinase

**Fig 4:**
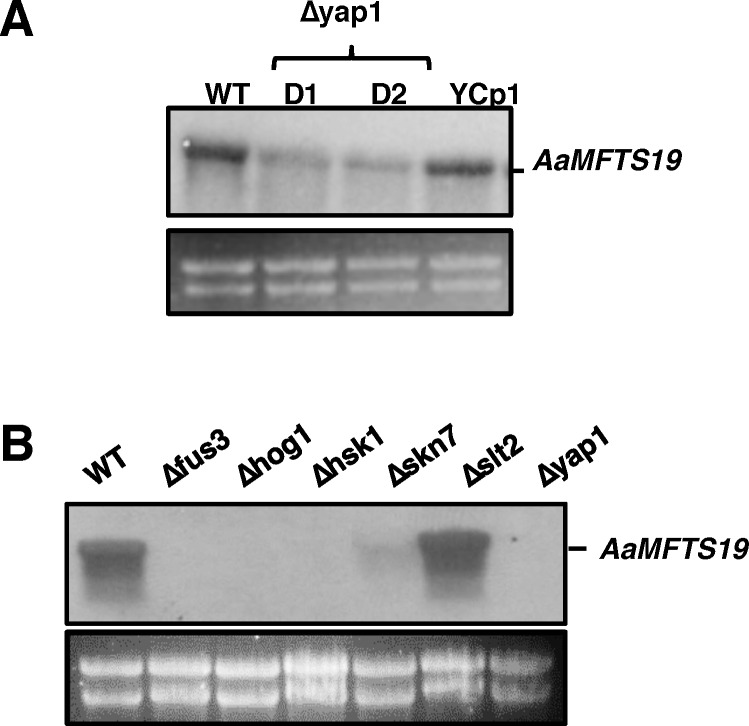
Northern blot analysis of *AaMFS19* in the wild-type (WT) and mutant strains of *Alternaria alternata*. **(A)** RNA was purified from WT, the mutant strains lacking Yap1 transcription regulator (Δyap1-D1 and D2), and the YCp1 strain re-acquiring and expressing a functional copy of *Yap1*. **(B)** RNA was purified from fungal strains impaired for a Fus3 MAP kinase (Δfus3), a Hog1 MAP kinase (Δhog1), a histidine kinase (Δhsk1), a Skn7 response regulator, and a Slt2 MAP kinase (Δslt3). Fungal strains were grown on a layer of cellophane overlaid on PDA for 3 days. RNA was purified, electrophoresed on a formaldehyde-containing gel, blotted to membrane, and hybridized with an *AaMFS19*-specific probe. Ethidium bromide (EtBr)-stained rRNA is shown to ensure equal loading of the samples.

Northern blot hybridization revealed further that the accumulation of the *AaMFS19* transcript was decreased in fungal strains defective for the stress-responsive transcription regulator Yap1 (Fig 4A), consistent with previous findings [10]. The *AaMFS19* transcript was detected at similar levels in the wild-type and the *Yap1* complementation strains. Inactivation of a Hog1 MAP kinase-coding gene also resulted in a decrease of the *AaMFS19* transcript (Fig 4B). Fungal strains inactivated at a “two-component” histidine kinase (Hsk1)-coding gene accumulated much lower levels of the *AaMFS19* transcript than wild-type. Similar reduction of the *AaMFS19* transcript was also observed in fungal strains impaired for a Fus3 MAP kinase or a Skn7 response regulator. However, mutation at a Slt2 MAP kinase did not affect the expression of *AaMFS19*.

### *AaMFS19* plays a role in fungal virulence

**Fig 5:**
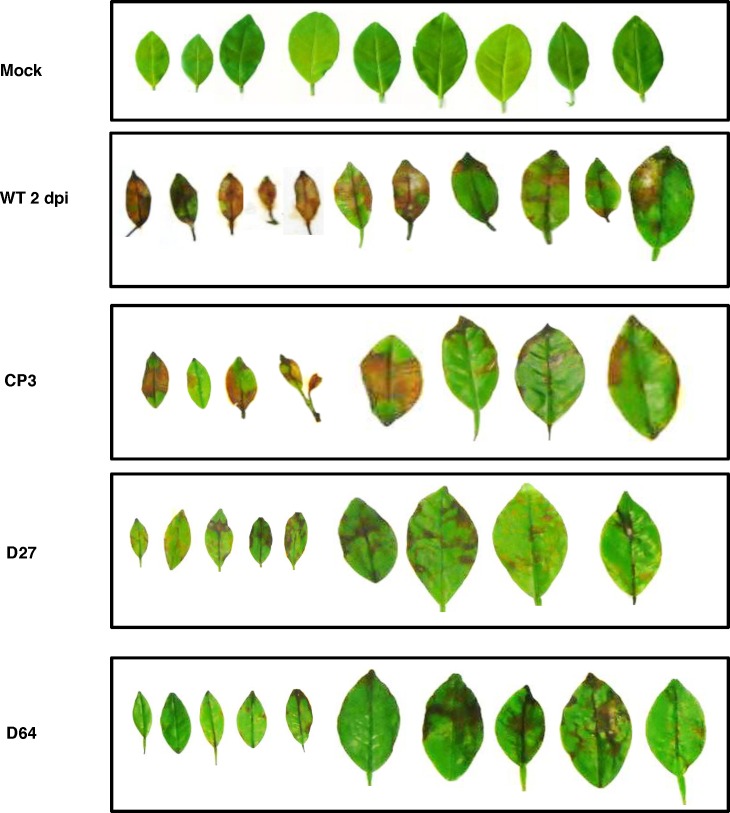
Virulence assays on detached calamondin leaves. Conidial suspension (10^4^ conidia/ml) collected from the wild type, the strains lacking *AaMFS19* (D27 and D63), and the CP3 strain expressing a copy of *AaMFS19* were sprayed uniformly onto detached calamondin leaves. The mock controls were treated with water only. The leaves were incubated in a moisture chamber for lesion development. Photos were taken 2 days post inoculation (dpi). Experiment was repeated three times with at least 10 leaves. Only representative replicates are shown.

Pathogenicity tests assayed on detached calamondin leaves using a point inoculation (10 μl, 10^5^ conidia/ml) method revealed no significant differences in lesions induced by the wild-type, the *AaMFS19* mutant, and the CP3 complementation strains (data not shown). However, pathogenicity assessed further on detached citrus leaves sprayed uniformly with conidial suspension (10^5^ conidia/ml) revealed a reduction of necrotic lesions induced by the *AaMFS19* mutants, as compared to those induced by the wild-type and the Cp3 strains (Fig 5).

### Pleiotropic effects of TIBA and CHP on *A*. *alternata*

**Fig 6:**
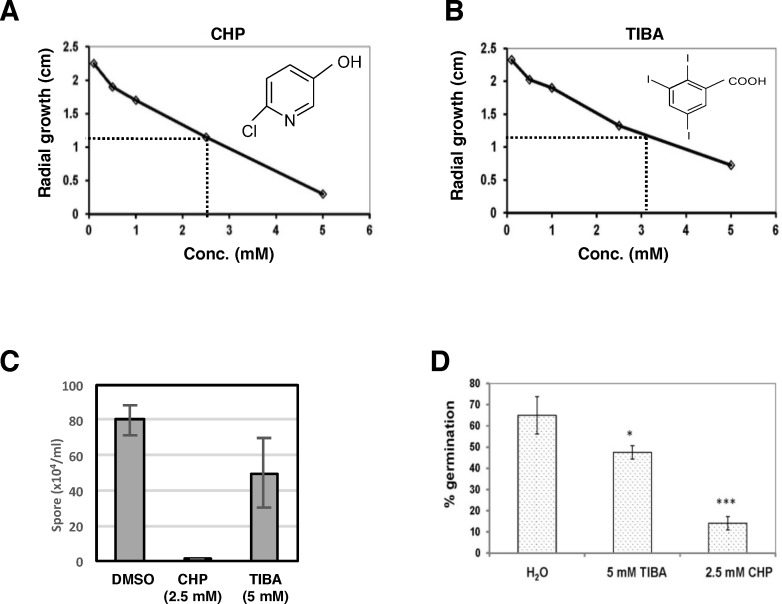
Toxicity of 2-chloro-5-hydroxypyridine (CHP) and 2,3,5-triiodobenzoic acid (TIBA) to *Alternaria alternata*. Wild-type was grown on PDA amended with **(A)** CHP and **(B)** TIBA at different concentrations and colony diameter was measured 5 days post inoculation. **(C)** Wild-type was cultured on PDA amended with 0.1% dimethyl sulfoxide (DMSO), 2.5 mM CHP, or 6 mM TIBA for 5 days and conidia were harvested and examined microscopically. **(D)** Conidia were germinated on glass slide, incubated in a moist chamber for 6 h, and examined microscopically. The data presented are the mean and standard deviation of two independent experiments with two biological replicates.

**Fig 7:**
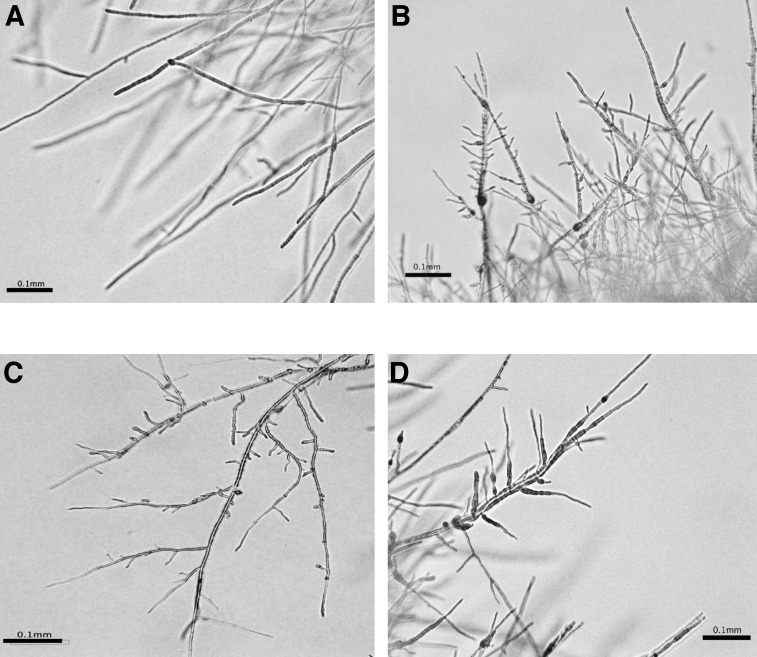
2-chloro-5-hydroxypyridine (CHP) and 2,3,5-triiodobenzoic acid (TIBA) affects hyphal elongation of *Alternaria alternata*. Wild-type was grown on **(A)** PDA, **(B) (C)** PDA amended with CHP, and **(D)** with TIBA for 3 days. Hyphae were examined microscopically.

As described above, *A*. *alternata* impaired at *AaMFS19* increased sensitivity to both 2-chloro-5-hydroxypyridine (CHP, 3 mM) and 2,3,5-triiodobenzoic acid (TIBA, 5 mM) compared to wild-type. The toxicity of CHP and TIBA to *A*. *alternata* remains elusive. The wild-type strain of *A*. *alternata* displayed sensitivity to both compounds in a dosage-dependent manner (Fig 6A and 6B). The effective concentrations of TIBA and CHP resulting in 50% inhibition (IC_50_) of *A*. *alternata* were around 3.2 and 2.6 mM, respectively. Treatment of *A*. *alternata* with CHP or TIBA resulted in a marked reduction in the production and germination of conidia (Fig 6C and 6D). Treatment of *A*. *alternata* with CHP or TIBA also resulted in an increase of hyphal branching (Fig 7).

## Discussion

*AaMFS19* encoding a MFS transporter was first identified from a cDNA library prepared from the wild-type strain of *A*. *alternata* after subtraction with cDNA from a *Yap1* mutant [10]. In the present study, AaMFS19 containing 12 transmembrane helixes has been shown to be required for cellular resistance to cumyl H_2_O_2_, KO_2_, and many singlet oxygen-generating compounds including eosin Y, rose Bengal, hematoporphyrin, methylene blue, and cercosporin [25]. Although toluidine blue (TB) has also been known to generate singlet oxygen [26], the compound promoted radial growth of the *AaMFS19* mutant strains (D27 and D64). Furthermore, accumulation of the *AaMFS19* gene transcript was shown to be regulated by the stress-responsive regulators Yap1, Hog1, and Skn7, which confirms further the involvement of *AaMFS19* in resistance to ROS-producing compounds. The results also indicate that ROS resistance in *A*. *alternata* is, at least in part, mediated by membrane-bound transporters. Despite the fact that the function of *AaMFS19* is associated with ROS resistance, it seems unlikely that AaMFS19 is specifically functioning at ROS, as it is not required for resistance to H_2_O_2_ or other ROS-generating compounds such as diamide, menadione, *tert*-butyl hydroperoxide, and toluidine blue. In the budding yeast *Saccharomyces cerevisiae*, the involvement of MFS transporters in multidrug resistance and oxidative stress response, likely either through metabolic regulation or change of the plasma membrane compositions, has also been observed [27–29]. The expression of many MFS transporter coding genes has been found to be regulated by oxidative stress-related transcription factors including Yap1, Msn2, Msn4, and Sfp1 [2].

It appears that *AaMFS19* is required for resistance to coppers (CuCl_2_ and CuSO_4_) and copper fungicides, indicating that AaMFS19 could serve as a copper exporter. Experimental results also have shown that fungal strains with *AaMFS19* deficiency increase sensitivity to clotrimazole and fludioxonil fungicides. The diversity of AaMFS19 function in cellular tolerance to fungicides, oxidants, and xenobiotics indicates the important role of active efflux systems in multidrug resistance. The results also show that AaMFS19 has a general function capable of exporting structurally diverse chemicals. Fungi have many membrane-bound transporters, all of which could function synergistically against toxic chemicals [2,30,31]. Thus, it is not surprising that AaMFS19 plays a moderate role in multidrug resistance.

Pyridine (Fig 6A) composed of an aromatic ring with five carbons and one nitrogen atom can be found in many natural products and could react with H_2_O_2_ to form superoxide and hydroxyl radicals in the presence of Cu^2+^ [32,33]. Pyridine and its derivatives are essential parts of RNA, DNA, NADP/NADPH, flavin nucleotides (FAD/FADH), ATP, and GTP in the biological systems. The compound 2,3,5-triiodobenzoic acid (Fig 6B) is an inhibitor of indole-3-acetic acid (IAA) transportation and often used as an herbicide [34,35]. However, their toxicity toward fungal pathogens has never been determined. In the present study, we have shown that TIBA and CHP are toxic to the citrus fungal pathogen *A*. *alternata* by suppressing radial growth and by reducing conidial formation and germination. Wild-type strain treated with either CHP or TIBA displayed an abnormal formation of hyphae, frequently producing short branches. Both CHP and TIBA also interfere with the expression of the *AaMFS19* gene in *A*. *alternata*, even though AaMFS19 plays a role in CHP and TIBA resistance. CHP and, to a lesser extent, TIBA also impact the expression of actin and β-tubulin coding genes. Similar transcriptional inhibition is also observed in *A*. *alternata* treated with fludioxonil, vinclozolin, and clotrimazole fungicides as well as with H_2_O_2_. However, CHP, TIBA, and fungicides have no effects on the expression of *Gpx3* encoding a glutathione peroxidase, indicating that CHP and TIBA selectively suppress gene regulation.

Mutational inactivation of *AaMFS19* resulted in two fungal strains that increase sensitivity to CHP and TIBA. Expressing a functional copy of *AaMFS19* in one of the mutants restored wild-type levels of resistance, confirming the involvement of *AaMFS19* in cellular resistance to CHP and TIBA. However, AaMFS19 is apparently unable to fully protect *A*. *alternata* from the toxicity of CHP and TIBA. AaMFS19 likely acts to efflux CHP and TIBA from the fungal cells and prevent an excessive accumulation of these toxic chemicals. It is also likely that in addition to multiple drug and stress resistance, AaMFS19 could play an indirect role in stress resistance. A correlation between multidrug resistance and membrane lipid homeostasis has been established in fungi [36,37]. Nevertheless, AaMFS19 is able to permit normal grow at lower concentrations of chemicals. *Alternaria alternata* mutant impaired for *Yap1* increased hypersensitivity to TIBA and CHP [10] and thus Yap1-mediated resistance to these compounds is likely operated through activation of *AaMSF19*.

In addition to *Yap1*, *A*. *alternata* strains impaired for *Hsk1*, *Skn7*, *Hog1*, or *Fus3* increased sensitivity to TIBA and CHP [12,15,38] and decreased the expression of *AaMFS19*. The results suggest that Hsk1, Skn7, Hog1, and Fus3-mediated signaling pathways conferring TIBA and CHP resistance are closely associated with the function of AaMFS19. However, increased cellular sensitivity to fludioxonil fungicide seen in the *AaMFS19* mutants is unlikely mediated by Hsk1, Skn7, and Hog1, because mutation of *Hsk1*, *Skn7*, or *Hog1* results in fungi that are resistant to fludioxonil [12,15]. As with CHP and TIBA, fludioxonil, vinclozolin, and clotrimazole fungicides suppress the expression of genes encoding AaMFS19, actin, and β-tubulin. In addition, Hsk1, Skn7, and Hog1 play an important role in osmotic stress induced by salts and sugars while AaMFS19 plays no role at all (Pei-Ling Yu, *unpublished*). Increased sensitivity to copper fungicides seen in the *AaMFS19* mutants is not regulated by Fus3 because inactivation of *Fus3* leads to resistance to copper fungicides in *A*. *alternata* [38]. The expression of *AaMFS19* is apparently differentially regulated in response to different environmental stimuli. Our results demonstrate that AaMFS19 plays a complex role in physiological and pathological functions.

Pathogenicity tests reveal that AaMFS19 plays a role in fungal virulence because gene deletion mutants of *AaMFS19* are reduced in their ability to induce necrotic lesions on detached calamondin leaves after uniformly spraying with conidial suspensions. The results suggest an important biological role of AaMFS19 during pathogenesis *in planta*. Whether or not AaMFS19 plays a role in resistance to natural plant toxins awaits to be determined. Lastly, because the *AaMFS19* mutant strains also increase sensitivity to many ROS-generating compounds, the results derived from the present study confirm previous finding that the ability to deal with ROS plays an important role on *A*. *alternata* pathogenesis in citrus.

## Material and Methods

### Fungal strains and growth conditions

The wild-type strain (EV-MIL31) of *A*. *alternata* was cultured from diseased leaves of Minneola tangelo and has been previously characterized [14,18]. Fungal strains defective for a Yap1 regulator (Δyap1), a Skn7 response regulator (Δskn7), a high osmolarity glycerol (Δhog1) mitogen-activated protein kinase (MAPK), a cell wall integrity MAPK (Δslt2), a Fus3 MAPK (Δfus3), and a “two component” histidine kinase (Δhsk1) were generated from the EV-MIL31 strain in previous studies [11,12,15,38,39]. YCp1 strain was previously created by expressing a functional copy of *Yap1* in a Δyap1 mutant [11]. Fungal strains were cultured on potato dextrose agar (PDA; Difco, Sparks, MD) at 28°C with constant fluorescent light. For DNA and RNA purification, *Alternaria* strains were cultured on PDA covered with a sterile cellophane membrane. For protoplast isolation, fungi were cultured in potato dextrose broth (PDB) on a shaker for 3 to 4 days. Fungal transformants were recovered from a regeneration medium [40] amended with hygromycin (250 μg/ml, Roche Applied Science, Indianapolis, IN) or sulfonylurea (5 μg/ml, chlorimuron ethyl; Chem Service, West Chester, PA).

### Sensitivity tests

Chemical sensitivity was assayed on PDA plates supplemented with a test compound. Fungal hyphae and conidia from 5 to 7 day-old culture were picked with sterile toothpicks and transferred onto the test medium. Fungal cultures were incubated at 28°C in constant fluorescent light at intensity of 40 μE/m/ sec and colony radius was measured at 5 to 9 days. Each treatment contained four replicates and all experiments were performed at least two times. The difference of radial growth of the disruption mutants relative to the wild-type cultured on the same plate was measured. Percentage change was determined by dividing the relative difference of the growth by the wild-type growth and then multiplying by 100. All test chemicals were purchased from Sigma-Aldrich (St. Louis, MO, USA).

### Production and germination of conidia

Fungal mycelium was ground in sterile water using a disposable pestle and sprayed evenly onto PDA. Plates without sealing were placed in a plastic box and incubated at 28°C in constant fluorescent light for 3 to 5 days. Conidia were harvested by scrapping with sterile water and low-speed centrifugation (5000 x g) and examined microscopically with the aid of a hemacytometer. Conidia were germinated on glass slide incubated in a moist chamber and examined with a Leitz Laborlux phase contrast microscope (Leitz Wetzlar, Germany). Germination of conidia was determined by placing them on glass slides and incubating in a plastic box for 6 h and examined microscopically.

### Cloning, targeted gene disruption and genetic complementation

The *A*. *alternata* major facilitator superfamily (AaMFS) gene fragment (clone #19; accession no. GS597470) was previously identified from a wild-type cDNA library after subtraction with that of a *YAP1* null mutant [10]. The full-length *AaMFS19* gene sequence was identified from the completed genome sequence of *A*. *alternata* [41] and was amplified by PCR with the primers from genomic DNA. Fungal DNA was purified using a DNeasy Plant kit (Qiagen). Open reading frame (ORF) and exon/intron positions were predicted using Softberry gene-finding software.

*AaMFS19* gene was inactivated by inserting a bacterial phosphotransferase B gene (*HYG*) cassette under the control of the *Aspergillus nidulans trpC* gene promoter and terminator, conferring resistance to hygromycin in the genome of *A*. *alternata*. Truncated but overlapping *HYG* fragments (M13R19/hyg3 and hyg4/M13F) were amplified by PCR and fused with truncated *AaMFS19* fragments by two-round PCR (S1 Fig). A 5’*AaMFS19*::5’*HYg* fusion fragment (1.5 + 1.2 kb) was amplified by two-round PCR with the primers 19F, M13R19, M13R and hyg3. A 3’*AaMFS19*::3’*hYG* fusion fragment (1.3 + 1.8 kb) was amplified with primers hyg4, M13F, M13F19N, and 19R. The primers M13R19 and M13F19N contain the oligonucleotides completely complementary to the sequence of primers M13R and M13F, respectively. Amplicons (10 μl each) were mixed and introduced into fungal protoplasts prepared from the EV-MIL31 strain using polyethylene glycol and CaCl_2_ as previously described [40,42]. Fungal transformants growing on a regeneration medium amended with 250 μg/ml hygromycin (Roche Applied Science) were selected and examined by PCR with an *AaMFS19*-specific primer pairing with a *HYG*-specific primer as indicated in S1 Fig. The 473F and 5KR primers that are not present in the split marker fragments, respectively, were paired with the hyg3 and hyg4 primers and used to examine for the occurrence of homologous integration within *AaMFS19*. A 3.0-kb fragment was amplified with the primers 473F and hyg3 from genomic DNA prepared from transformants D27 and D64 and no product was amplified from that of wild-type. A 2.7-kb fragment was amplified with the primers 5KR and hyg4 from genomic DNA of D27 and D64 but not wild-type.

For genetic complementation, a full-length *AaMFS19* fragment under the control of its native promoter (3.8 kb) was amplified by PCR with two *AaMFS19*-specific primers (19F and 19R) and co-transformed with the pCB1532 plasmid carrying a sulfonylurea-resistant (*Sur*) gene [43] into protoplasts of an *AaMFS19* mutant (D27). Transformants appearing from medium amended with 5 μg/ml sulfonylurea were tested for restoration of cellular resistance to 3 mM 2-chloro-5-hydroxypyridine (CHP).

### Gene expression

Gene expression was assessed by Northern-blot hybridization and semi-quantitative RT-PCR. Fungal strains were grown on PDA covered with a layer of cellophane for 3 to 5 days. Fungal RNA was isolated from mycelium using a TriZol reagent (Invitrogen, Carlsbad, CA). For Northern-blot hybridization, RNA was electrophoresed in a formaldehyde-containing agarose gel, blotted onto a nylon membrane, and hybridized to an *AaMFS19* DNA probe. The probe was amplified and labeled with a digoxigenin (DIG)-11-dUTP by PCR with the *AaMFS19* gene-specific primers according to the manufacturer’s recommendation (Roche Applied Science). The probe was detected by an immunological assay using CSPD as a chemofluorescent substrate for alkaline phosphatase.

For medium shift experiments, the wild-type strain was cultured on PDA covered with a layer of cellophane for 2 days. The cellophane membranes containing fungal mycelium were lift and transferred onto a PDA supplemented with 0.1% dimethyl sulfoxide (DMSO), 5 mM 2,3,5-triiodobenzoic acid (TIBA), 2.5 mM CHP, 10 μM vincozolin, or 80 μM clotrimazole. After incubation for an additional 24 h, mycelium was harvested and subjected to RNA isolation. First-strand cDNA was synthesized using a MMLV High Performance Reverse Transcriptase (Epicentre) and used for PCR amplification with *AaMFS19*-specific primers. Amplicons were electrophoresed in 1% agarose gel and stained with ethidium bromide.

### Virulence assays

Fungal virulence was assessed on detached calamondin (*Citrus mitis* Blanco) leaves inoculated by placing 10 μl of conidial suspension (10^5^ conidia per ml) on each spot as described previously [14,16,17]. Alternatively, detached leaves were uniformly sprayed to run-off with conidial suspensions. The inoculated leaves were incubated in a plastic box for lesion formation. Each fungal strain was tested on at least 10 leaves and experiments were repeated three times.

### Ethics Statement

No ethical permissions were required for this work which involved no experimentation involving animals or human samples.

## Supporting Information

S1 Fig Targeted disruption of *AaMFS19* using a split marker approach and PCR confirmation of disruption. (DOCX)

S2 Fig *AaMSF19* deletion mutants show wild-type resistance to H_2_O_2_, *tert*-butyl hydroperoxide, diamide, and menadione. (DOCX)
