# Supplementary material for: A Major Facilitator Superfamily Transporter-Mediated Resistance to Oxidative Stress and Fungicides Requires Yap1, Skn7, and MAP Kinases in the Citrus Fungal Pathogen Alternaria alternata
Source: PLoS One. 2017 Jan 6;12(1):e0169103. doi: 10.1371/journal.pone.0169103 (PMC5218470; doi:10.1371/journal.pone.0169103)
Supplement: S1 Fig — (DOCX) [file pone.0169103.s001.docx]

**Funding:** This research was supported by a grant from the Ministry of Science and Technology of Taiwan (MOST103-2313-B-005-044-MY2 and MOST105-2313-B-005-010-MY3) to KRC. The funder had no roles in experimental design, data collection and analysis, decision to publish, or preparation of the manuscript.

**Supporting Information**

**S1 Fig. Targeted disruption of *AaMFS19* using a split marker approach.**

**A.** Schematic depiction of generation of truncated but overlapping hygromycin phosphotransferase gene (*HYG*) under control by the *Aspergillus nidulans* *trpC* promoter (P) and terminator (T) within *AaSSK1*. Oligonucleotide primers used to amplify each fragment are indicated.

**B.** Image of DNA fragments amplified from genomic DNA of wild type (WT) and transformants with the primer 473F paired with hyg3.


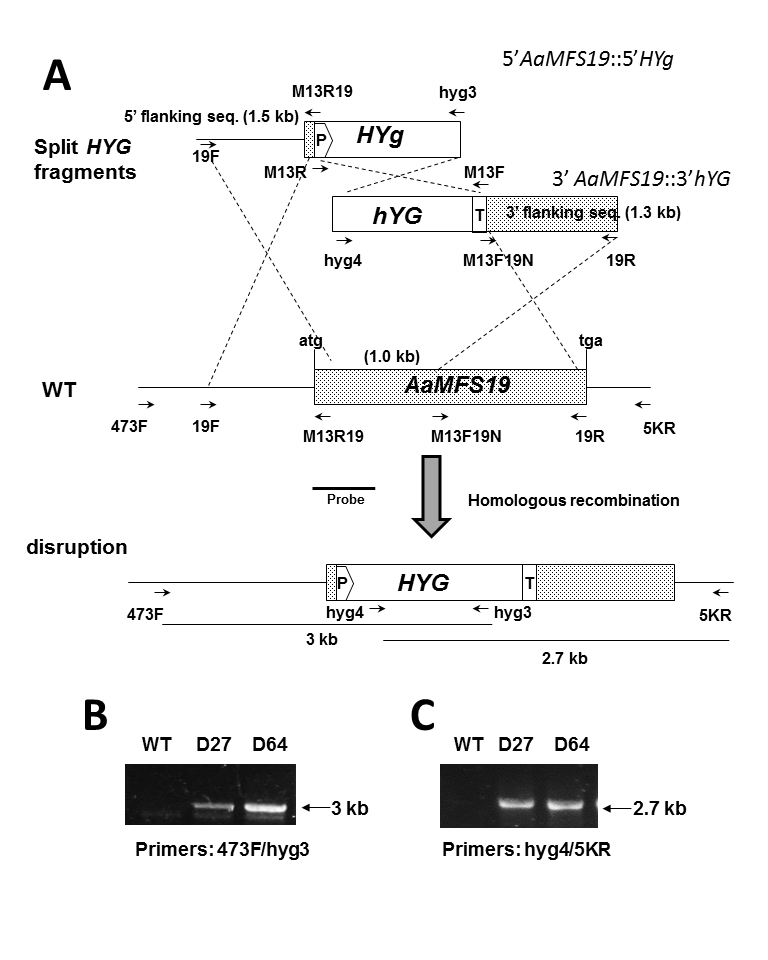
**C.** Image of DNA fragments amplified from genomic DNA of wild type (WT) and transformants with the primer hyg4 paired with 5KR, indicating that *AaMFS19* is deleted in transformants D27 and D64.
