# Supplementary material for: A Major Facilitator Superfamily Transporter-Mediated Resistance to Oxidative Stress and Fungicides Requires Yap1, Skn7, and MAP Kinases in the Citrus Fungal Pathogen Alternaria alternata
Source: PLoS One. 2017 Jan 6;12(1):e0169103. doi: 10.1371/journal.pone.0169103 (PMC5218470; doi:10.1371/journal.pone.0169103)

**Funding:** This research was supported by a grant from the Ministry of Science and Technology of Taiwan (MOST103-2313-B-005-044-MY2 and MOST105-2313-B-005-010-MY3) to KRC. The funder had no roles in experimental design, data collection and analysis, decision to publish, or preparation of the manuscript.

**Supporting Information**

**S2 Fig. *AaMSF19* deletion mutants show wild-type resistance to H2O2, *tert*-butyl hydroperoxide, diamide, and menadione.**

Images of the wild-type (WT), the ∆AaMSF19 deletion mutants (D27 and D64), and the complementation strain (Cp3) grown on potato dextrose agar (PDA) amended with different chemicals as indicated for 5 days.


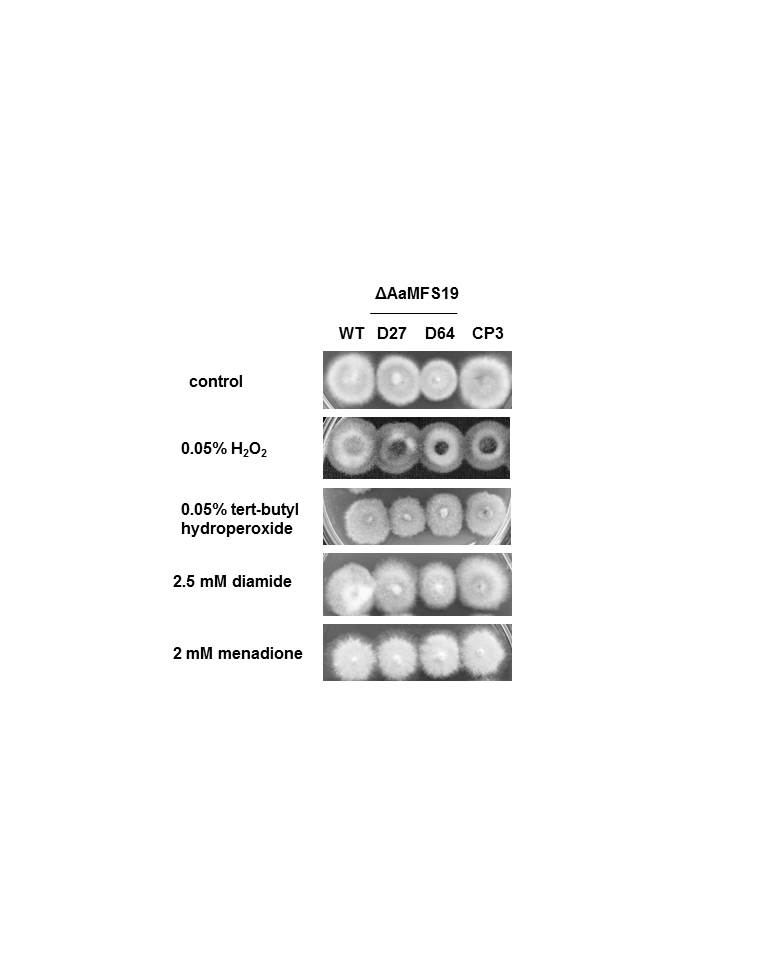

Supplement: S2 Fig — (DOCX) [file pone.0169103.s002.docx]
